# Phylogeny and taxonomy of *Cinnamomum* (Lauraceae)

**DOI:** 10.1002/ece3.9378

**Published:** 2022-10-01

**Authors:** Zhi Yang, Bing Liu, Yong Yang, David K. Ferguson

**Affiliations:** ^1^ Co‐Innovation Center for Sustainable Forestry in Southern China College of Biology and the Environment, Nanjing Forestry University Nanjing China; ^2^ State Key Laboratory of Systematic and Evolutionary Botany Institute of Botany, Chinese Academy of Sciences Beijing China; ^3^ Department of Paleontology University of Vienna Vienna Austria

**Keywords:** *Camphora*, *Cinnamomum*, Lauraceae, new combinations, phylogeny, taxonomy

## Abstract

Taxonomy of *Cinnamomum* Schaeff. of Lauraceae remains problematic because recent phylogenetic studies have suggested that this genus is not monophyletic. In this study, we assembled three sequence matrices including plastomes (datamatrix I), nrITS sequences alone (datamatrix II), and nrITS plus plastid *psb*A‐*trn*H sequences (datamatrix III) of the *Cinnamomum*‐*Ocotea* complex of Lauraceae and conducted a new phylogenetic study with thusfar the most extensive species sampling of the *Cinnamomum*‐*Ocotea* group. We determined that the Old World *Cinnamomum* is diphyletic: sect. *Camphora* Meisn. is sister to *Sassafras* J.Presl and sect. *Cinnamomum* is sister to the African *Kuloa* Trofimov & Rohwer. A recent study indicated that characters of leaf micromorphological anatomy can define the two clades: one possessing reticulate periclinal and the other having non‐reticulate periclinal walls. As result, we divided the genus *Cinnamomum* of Lauraceae into two genera, i.e., *Cinnamomum* and *Camphora* Fabr. The generic name *Cinnamomum* is retained for those species mainly having reticulate periclinal epidermal cell walls, inconspicuous non‐perulate terminal buds and usually tripliveined leaves; the oldest generic name, *Camphora*, is applied to the second group which contains those species mainly possessing non‐reticulate periclinal epidermal cell walls, prominent perulate terminal buds and pinnately‐veined leaves. A census of the species and their type specimens listed under *Cinnamomum* in Asia resulted in the transfer of 18 species to *Camphora*, including 15 new combinations.

## Graphical abstract

Cinnamomum is diphyletic according to a new phylogeny using nrITS and psbA‐trnH sequences. As a result, we classify the genus Cinnamomum into two genera, i.e., Camphora and Cinnamomum.

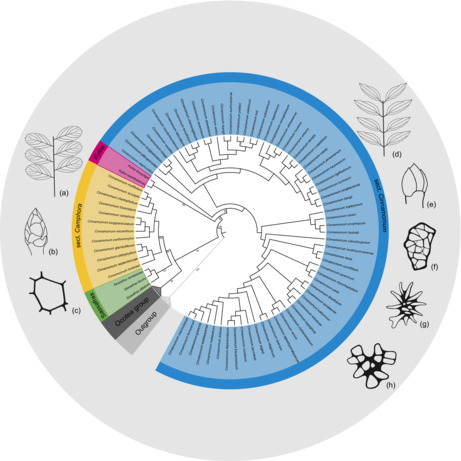

## 1 INTRODUCTION

The Lauraceae constitute the largest family of the order Laurales which is sister to the Magnoliales (The Angiosperm Phylogeny Group, 2016). This family contains more than 3000 species in ca. 55 genera that are mostly woody and widely distributed in the pantropics with tropical America and Australasia as the diversity centers (Renner, 2011). Taxonomy of the Lauraceae has been notorious due to imperfectly known species with either flower or fruit characters unknown, overlapping variation and parallel evolution of morphological characters, and poorly represented specimens in herbaria. Taxonomic delimitation of many generic groups has been controversial (van der Werff, 2001), e.g. *Beilschmiedia* group (Li et al., 2020; Yang et al., 2012), *Persea* group (Li et al., 2011; Mo et al., 2017), *Ocotea* group (Penagos Zuluaga et al., 2021; Trofimov et al., 2019, 2022), and *Cinnamomum* group (Gang et al., 2021; Huang et al., 2016; Rohde et al., 2017). The genus *Cinnamomum* Schaeff. is probably the most difficult one of the family Lauraceae because early researchers usually studied materials including detached leaves of uncertain origin that were mostly picked from immature plants (viz. cinnamon, Kostermans, 1970, 1985).

*Cinnamomum* is defined by a set of morphological characters including evergreen trees or shrubs, opposite and triplinerved or alternate and pinninerved leaves, paniculate‐cymose inflorescences, bisexual and trimerous flowers, nine fertile stamens in three whorls with the two outer whorls introrse and the innermost whorl of stamens extrorse with normally 4‐locular anthers, staminodes of the fourth whorl well developed, and fruits with a cupule (Lorea‐Hernández, 1996; Rohwer, 1993; van der Werff, 2001). These morphological characters imply controversial systematic positions of *Cinnamomum* (Bentham & Hooker, 1880; Kostermans, 1957, 1986; Meissner, 1864; Pax, 1891; Rohwer, 1993; Van der Werff & Richter, 1996). Kostermans (1957) classified the genus *Cinnamomum* together with *Ocotea* Aubl., *Actinodaphne* Nees, *Sassafras* J. Presl, *Umbellularia* Nutt. and *Dicypellium* Nees & Mart. in the subtrib. Cinnamomineae of the trib. Cinnamomeae. Rohwer (1993) classified the family Lauraceae into informal groups, and ascribed the genus *Cinnamomum* to the *Ocotea* subgroup of the *Ocotea* group together with *Neocinnamomum* H.Liou, *Aiouea* Aubl., *Endlicheria* Nees, *Rhodostemonodaphne* Rohwer & Kubitzki, *Ocotea*, *Nectandra* Rol. ex Rottb., *Pleurothyrium* Nees. Van der Werff and Richter (1996), however, included *Ocotea*, *Nectandra*, *Aniba* Aubl., *Licaria* Aubl., *Pleurothyrium* Nees, *Cinnamomum*, *Persea* Mill., *Phoebe* Nees, and *Dehaasia* Blume in the trib. Perseeae according to types of inflorescences.

Phylogenies based on nrITS, 26S, *trn*L‐*trn*F, *psb*A‐*trn*H, *trn*T‐*trn*L, and *rpl*16 sequences have suggested that the genus *Cinnamomum* belongs to the Cinnamomeae, but relationships of the Cinnamomeae were badly resolved with regard to Laureae (Chanderbali et al., 2001; Rohde et al., 2017). Based on a plastome phylogeny, Song et al. (2020) indicated a division of the family Lauraceae into six tribes with the genus *Cinnamomum* belonging to the trib. Laureae; that study did not sample the *Mezilaurus* group and the trib. Laureae is a mixture including the *Persea* group, the *Cinnamomum* group, and the *Litsea* group. Liu et al. (2021) suggested that the family Lauraceae should be classified into eight tribes according to recent phylogenetic studies, viz. Hypodaphnideae, Cryptocaryeae, Cassytheae, Neocinnamomeae, Caryodaphnopsideae, Perseeae, Cinnamomeae and Laureae (no samples of the *Mezilaurus* group), with the genus *Cinnamomum* included in the trib. Cinnamomeae.

**FIGURE 1:**
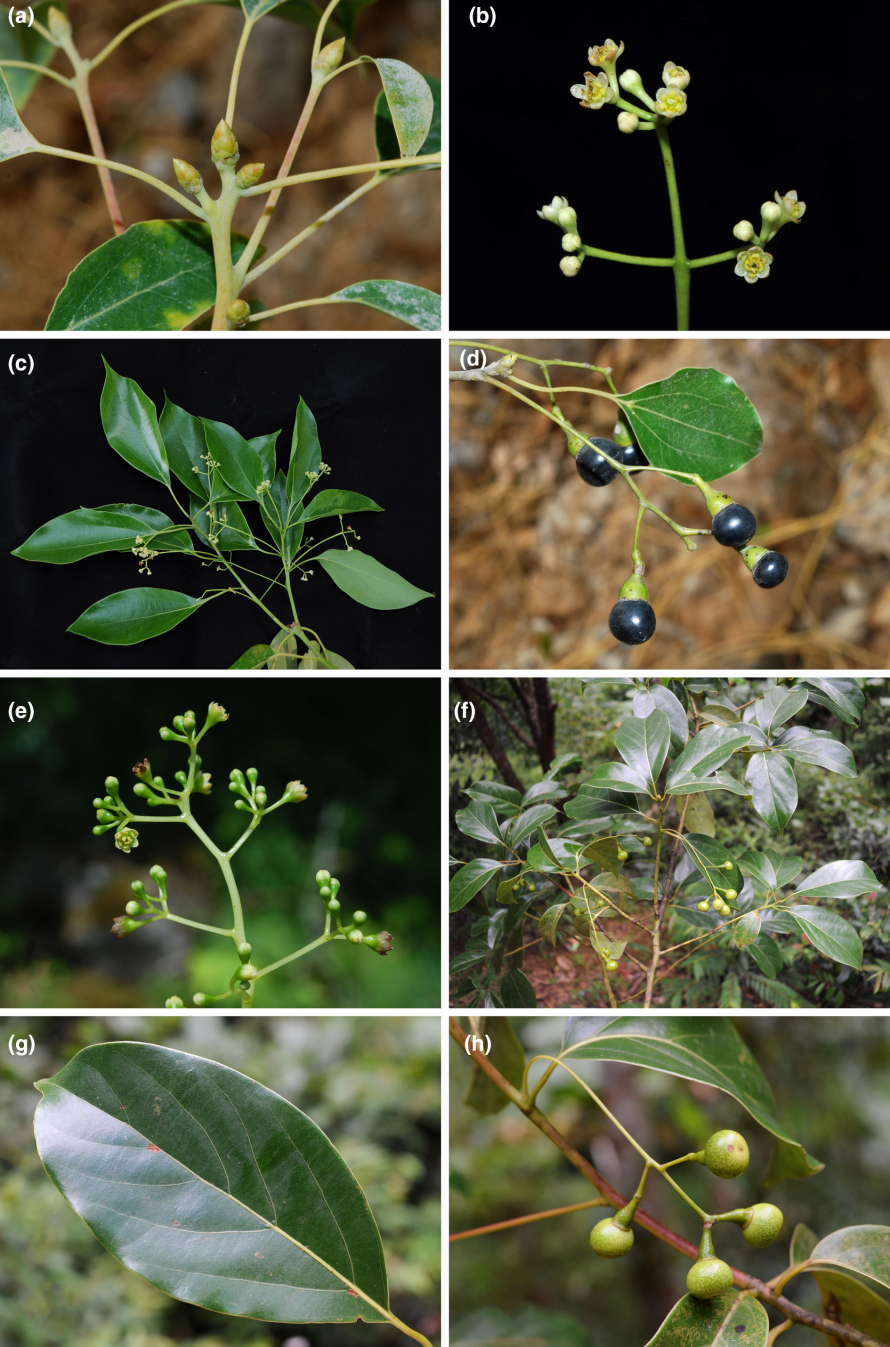
Morphology of *Cinnamomum* Schaeff. (a–d) *Cinnamomum camphora* (L.) J. Presl (= *Camphora officinarum* Nees); (e–h) *Cinnamomum glanduliferum* (Wall.) Meisn. (≡ *Camphora glandulifera* (Wall.) Nees).

**FIGURE 2:**
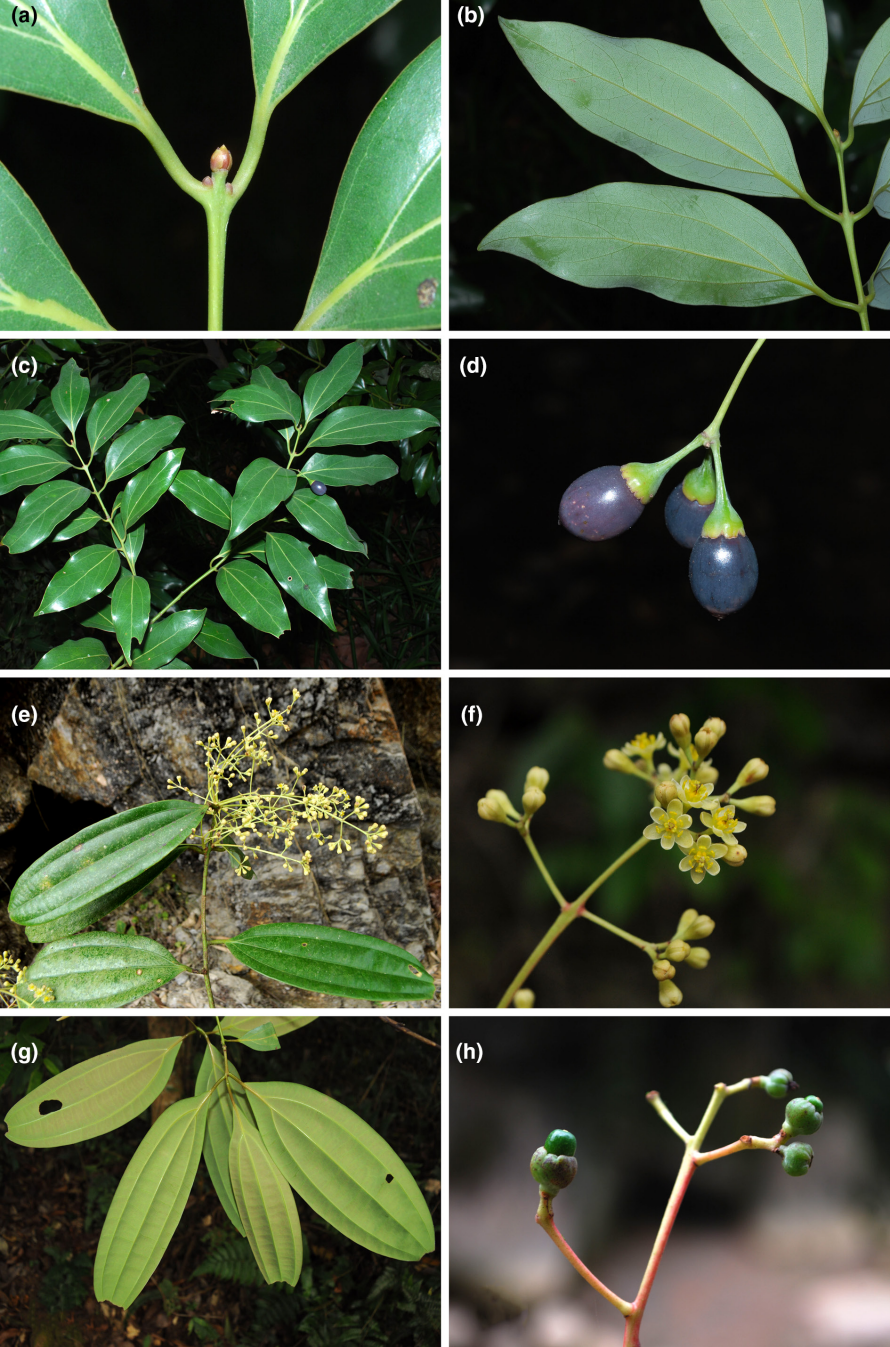
Morphology of *Cinnamomum* Schaeff. (a–d), *Cinnamomum japonicum* Sieb.; (e–h) *Cinnamomum bejolghota* (Buch.‐Ham.) Sweet.

The genus *Cinnamomum* was formerly considered to contain 350 species that are amphi‐Pacific (Lorea‐Hernández, 1996; Rohwer, 1993; van der Werff, 2001). Recent phylogenetic and taxonomic studies have transferred the American species to *Aiouea* (Rohde et al., 2017), so the remaining Old World *Cinnnamomum* now contains 247 species (https://powo.science.kew.org/taxon/urn:lsid:ipni.org:names:328262‐2#children). Species of *Cinnamomum* are usually subdivided into two groups: one group including species having alternate and pinnately veined leaves, domatia present in the axils of lateral veins and middle veins, and perulate terminal buds (Figure 1); and the other group containing species possessing opposite/subopposite and tripliveined leaves lacking domatia in the axils of lateral veins, and non‐perulate terminal buds (Figure 2; Lorea‐Hernández, 1996; Huang et al., 2016). The two groups are normally ranked as two sections, i.e. sect. *Camphora* Meisn. (syn.: sect. *Malabathrum* Meisn.) and *Cinnamomum* (Hooker, 1886; Kostermans, 1986; Li et al., 1982; Lorea‐Hernández, 1996; Meissner, 1864) Nees (1831, 1836), however, separated the two groups into two genera: *Camphora* and *Cinnamomum*. Besides the macromorphological differences, the two groups are also different in characters of the upper leaf epidermis: (1) the cell shape is regular in sect. *Camphora*, but irregular in sect. *Cinnamomum*; (2) the anticlinal wall is straight or nearly so in sect. *Camphora*, but sinuous in sect. *Cinnamomum*; and (3) the periclinal wall is smooth in sect. *Camphora*, but reticulate in sect. *Cinnamomum* (Gang et al., 2021).

Relationships of the *Cinnamomum*‐*Ocotea* complex have not been resolved. Trofimov and Huang et al. (2016) suggested that sect. *Cinnamomum* is sister to the Neotropical clade, whereas Rohde et al. (2017) indicated that sect. *Camphora* is sister to the Neotropical clade. Trofimov and Rohwer (2020) suggested that the Old World *Cinnamomum* is diphyletic or paraphyletic as well, with sect. *Cinnamomum* sister to *Kuloa* Trofimov & Rohwer and sect. *Camphora* sister to *Sassafras* J. Presl. Other recent studies considered the genus *Cinnamomum* as paraphyletic with regard to *Sassafras* (Liu et al., 2021; Song et al., 2020; Trofimov et al., 2022), which may be attributable to sampling bias, none of them included *Kuloa*. The phylogeny of *Cinnamomum* is thus not well resolved, so further phylogenetic studies are necessary to determine the monophyly of *Cinnamomum*, and the genus should be further subdivided if confirmed to be polyphyletic. As a result, our target here is to (1) reconstruct a resolved phylogeny of the *Cinnamomum*–*Ocotea* complex with a broad sampling of the genus *Cinnamomum* using separate and concatenated sequence matrices including plastomes, nrITS and *psb*A–*trn*H sequences, and (2) conduct a new synoptic taxonomy of sect. *Camphora* if the polyphyly of the genus is confirmed.

## 2 MATERIALS AND METHODS

A new phylogeny of the *Cinnamomum* group was conducted using all available complete chloroplast genome/plastomes (CPG) and two commonly used markers including nrITS and *psb*A–*trn*H in the family Lauraceae. To resolve the phylogeny of *Cinnamomum*, we also included species samples of the *Cinnamomum*‐*Ocotea* complex. *Alseodaphne semecarpifolia* Nees, *Machilus thunbergii* Siebold & Zucc., *Persea americana* Mill., and *Phoebe sheareri* Gamble were chosen as outgroups. Sequences were obtained from GenBank (https://www.ncbi.nlm.nih.gov/) and LCGDB (https://lcgdb.wordpress.com) (Appendix A, last search 22 March 2022), aligned in MAFFTT (Katoh et al., 2017) using Auto and Localpair model for CPG and the two markers respectively, then adjusted and edited manually in BioEdit (Hall, 1999). Ambiguously aligned fragments of CPG were removed with Gblocks using default setting (Talavera & Castresana, 2007) and gap sites of nrITS and *psb*A‐*trn*H sequences were deleted with trimAl using “‐automated1” (Capella et al., 2009). Totally, we assembled three matrices: complete plastome sequences (datamatrix I), nrITS (datamatrix II), and a datamatrix including nrITS and *psb*A‐*trn*H (datamatrix III). The two markers of datamatrix III were concatenated using PhyloSuite (Zhang et al., 2020). A best‐fit or partition model of all matrices was computed with ModelFinder (Kalyaanamoorthy et al., 2017). For phylogenetic studies, maximum likelihood (ML) analyses were conducted in IQ‐TREE (Nguyen et al., 2014), bootstrap values were obtained using Ultrafast Bootstrap for 5000 and 1000 times for the datamatrix I and other two datamatrices separately (Minh et al., 2013); Bayesian inferences (BI) were conducted in MrBayes (Ronquist et al., 2012) with the following parameters: generations: 12,000,000, sampling frequency: 6000, and burnin: 25.0%. Phylogenetic trees were viewed and edited in ITOL (Letunic & Bork, 2021), and improved in Adobe Illustrator 2020.

## 3 RESULTS

**FIGURE 3:**
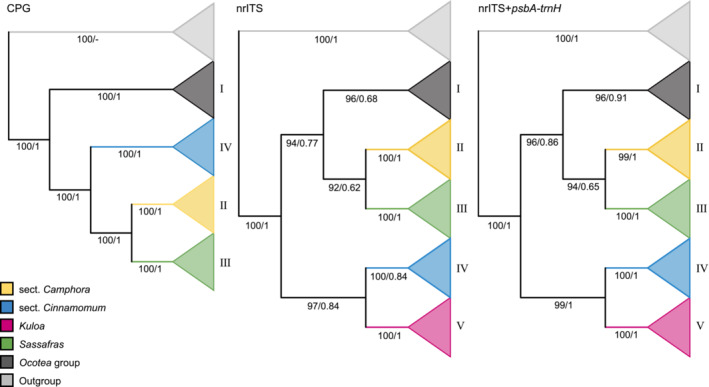
Phylogenetic trees of the *Cinnamomum*‐*Ocotea* complex based on three sequence datamatrices using maximum likelihood (ML) and Bayesian inference (BI). The support values of ultrafast bootstrap (UFBS ≥ 70%; on the left) and posterior probability (PP ≥ 70%; on the right) are shown below branches.

**FIGURE 4:**
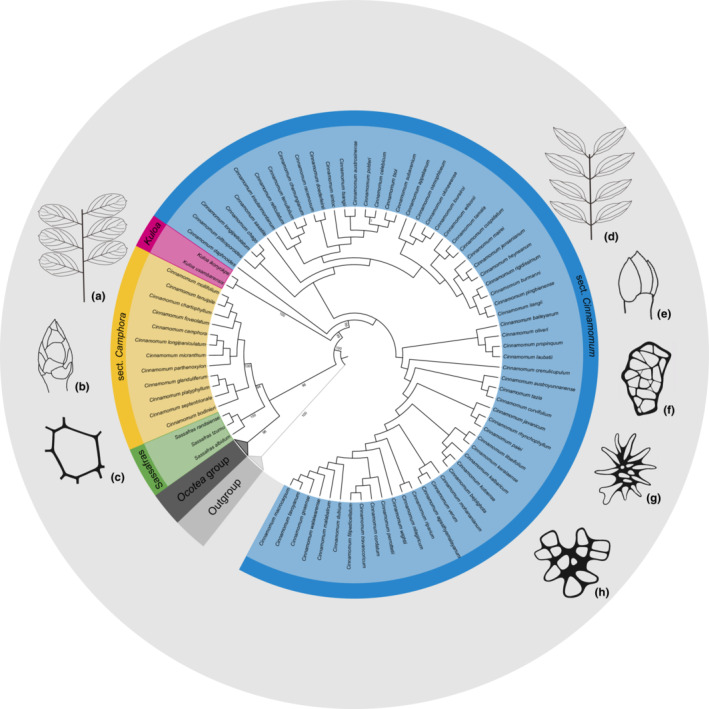
Maximum likelihood (ML) tree of the *Cinnamomum*‐*Ocotea* group using nrITS and *psbA*‐*trnH* data. Ultrafast bootstrap values of the outgroup and the five ingroup clades are shown on the tree. Illustrations display macro‐ and micromorphological characters of sect. *Camphora* s.s. (a–c) and *Cinnamomum* s.l. (d–h). (a) Alternate and pinnately veined leaves; (b) prominent perulate terminal buds; (c) non‐reticulate periclinal wall of the upper leaf epidermis; (d) opposite/subopposite and tripliveined leaves; (e) non‐perulate terminal bud; (f,g) reticulate periclinal wall of the upper leaf epidermis. The illustrations c, f, and g were published by Gang et al. (2021).

We finally obtained 38 plastomes, 324 nrITS and 238 *psb*A‐*trn*H sequences. Statistic data of the three datamatrices and their best‐fit models were listed in Table 1. Our plastome phylogeny (datamatrix I) contained four ingroup clades due to the lack of plastome sequences of *Kuloa* (Figure 3a). Our phylogenetic trees based on nrITS alone (datamatrix II) or nrITS plus *psb*A‐*trn*H (datamatrix III) resulted in five large clades (Figures 3b,c and 4): Clade I including the American genera of the *Cinnamomum*‐*Ocotea* complex; Clade II comprising sect. *Camphora* s.s. (excluding *C. chago* B.S.Sun & H.L.Zhao, *C. longipetiolatum* H.W. Li, and *C. saxatile* H.W. Li, here defined); Clade III containing the deciduous genus *Sassafras*; Clade IV including sect. *Cinnamomum* s.l. (including *C. chago*, *C. longipetiolatum*, and *C. saxatile* of sect. *Camphora* s.l.); and Clade V encompassing the African *Kuloa*. These phylogenetic trees based on different datamtrices gave rise to similar relationships of the five large clades, the genus *Cinnamomum* was polyphyletic, sect. *Camphora* s.s. was sister to *Sassafras*, and sect. *Cinnamomum* s.l. was sister to *Kuloa*. Relationships within the two sections of *Cinnamomum* were not resolved. To show morphological differences of the two groups of *Cinnamomum*, we mapped both macro‐ and micro‐morphological characters on the combined tree based on nsITS plus *psb*A‐*trn*H sequences (Figure 4).

**TABLE 1:** Statistics of sequence information for phylogeny of this study

Item	nrITS	nrITS *+ psb*A‐*trn*H	*psb*A‐*trn*H	Plastome
Aligned length (nt)	867	1511	644	152,510
Variable sites (nt)	458	743	285	3611
Parsimony informative sites (nt)	310	486	176	1690
V%	52.83%	49.17%	44.25%	2.37%
P%	35.76%	32.16%	27.33%	1.11%
Model (ML)	TIM + F + I + G4	Partitioned	–	K3Pu + F + I + G4
Model (BI)	GTR + F + I + G4	Partitioned	–	GTR + F + I + G4

## 4 DISCUSSION

Our new phylogenies using nrITS alone and nrITS plus *psb*A‐*trn*H sequences included thusfar the most extensive species sampling of the *Cinnamomum*‐*Ocotea* complex. We have identified five large clades including the *Ocotea* group (Clade I), sect. *Camphora* (Clade II), *Sassafras* (Clade III), sect. *Cinnamomum* (Clade IV), and *Kuloa* (Clade V). The phylogenetic position of the *Ocotea* group is different between our new phylogeny and a few previous studies (Huang et al., 2016; Rohde et al., 2017; Trofimov & Rohwer, 2020): the *Ocotea* group is sister to a clade including three subclades (sect. *Camphora*, sect. *Cinnamomum* and *Sassafras*) or to two subclades (sect. *Camphora* and *Sassafras*) in our new phylogeny but sister to a clade containing sect. *Cinnamomum* plus *Kuloa* (the African *Ocotea*) in Huang et al. (2016), and sister to sect. *Camphora* plus *Sassafras* in Trofimov and Rohwer (2020). Rohde et al. (2017) suggested additional relationships, i.e. *Sassafras* alone is sister to a large clade including the *Ocotea* group plus *Cinnamomum*, in the large clade the *Ocotea* group and sect. *Camphora* forms a subclade sister to sect. *Cinnamomum*; their phylogenetic trees possess very low bootstrap values. However, our plastome phylogenetic tree shows similar topology to that of Trofimov et al. (2022) that the *Ocotea* group is sister to a clade including sect. *Cinnamomum* and sect. *Camphora* plus *Sassafras*. Our new phylogenetic results clearly suggest that the Old World *Cinnamomum* species are diphyletic and represent two separate groups, which is consistent with recent studies using representative species sampling (Huang et al., 2016; Rohde et al., 2017; Trofimov & Rohwer, 2020). Our plastome phylogeny indicates that the genus *Cinnamomum* is paraphyletic with respect to *Sassafras*, which agrees with the result of Trofimov et al. (2022); this relationship is probably caused by the lack of sampling in the African *Kuloa* and the incongruence between cpDNA and nuclear DNA phylogenies; no plastomes of *Kuloa* are available at present. Taken together, we conclude that the genus *Cinnamomum* is diphyletic.

**FIGURE 5:**
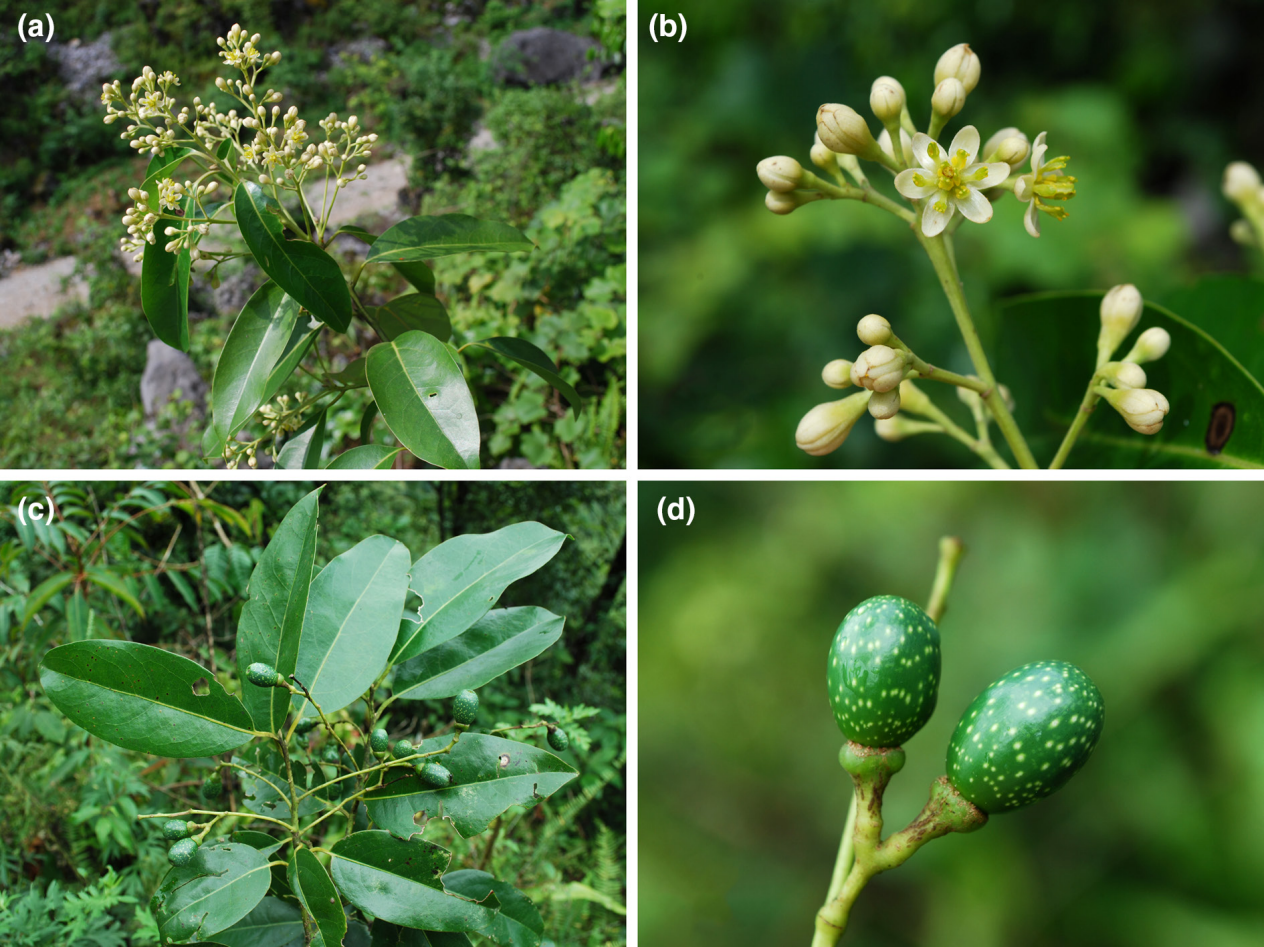
Morphology of *Cinnamomum saxatile* H.W. Li, an unusual species of *Cinnamomum* Schaeff. With pinnately veined leaves. (a) Inflorescence branch; (b), inflorescence; (c), infructescence branch; (d), fruit.

For a new classification, we also considered macromorphology and micromorphology. Macromorphological characters are largely consistent with the phylogenetic results, e.g. buds perulate or not, leaves alternate or opposite, pinnately veined or tripliveined, domatia presence in axil of lateral veins (Figure 5), except for *C. chago*, *C. longipetiolatum*, and *C. saxatile*. A recent study of leaf epidermal micromorphology in the Old World *Cinnamomum* species by Gang et al. (2021) found two types of micromorphology that were clade‐specific and highly predictive. The periclinal wall ornamentation coincides perfectly with the phylogenetic results seen here, i.e., sect. *Camphora* s.s. possessing a non‐reticulate periclinal wall and sect. *Cinnamomum* s.l. having a reticulate periclinal wall (Gang et al., 2021; and our study here). Considering the congruence of macromorphological, micromorphological and phylogenetic results, *Cinnamomum* as currently circumscribed is therefore best divided into two genera.

*Cinnamomum* sect. *Cinnamomum* s.l. embraces the generic type: *C. verum* J. Presl (syn.: *C. zeylanicum* Blume), and thus, should retain the generic name and a different generic name be given to sect. *Camphora* s.s. under Art. 10.8 of the *Shenzhen Code* (Turland et al., 2018). There are several generic names listed under synonymy for *Cinnamomum* s.l. (e.g., Li et al., 1982, 2008; Rohwer, 1993), including *Camphora* Fabr., *Cecidodaphne* Nees, *Parthenoxylon* Blume and *Temmodaphne* Kosterm. *Cecidodaphne* Nees is based on *C. glaucescens* Nees (≡*Cinnamomum glaucescens* [Nees] Hand.‐Mazz.) and should be considered as a synonym of *Cinnamomum* in the narrow sense, because the type species bears tripliveined leaves and belongs to sect. *Cinnamomum*. Similarly, the type specimen of *Temmodaphne* (*T. thailandica* Kosterm.) also has triplinerved, sub‐opposite leaves (Kostermans, 1973) suggesting that it also belongs in *Cinnamomum* s.str. *Parthenoxylon* is based upon *P. porrectum* (Roxb.) Blume, and treated as a synonym of *C. parthenoxylon* (Jack) Meisn. by the *Flora of China* (Li et al., 2008), so is potentially available for the non‐*Cinnamomum* clade. However, *Camphora* (Fabricius, 1759) has priority over *Parthenoxylon* (Blume, 1849–1851). As a result, we transfer those species usually with alternate and pinnately veined leaves, domatia present in axil of lateral veins, perulate buds, and non‐reticulate periclinal walls to *Camphora*.

## 5 TAXONOMIC TREATMENT

### 5.1 Cinnamomum

Schaeff., Bot. Exped. 74. Oct‐Dec 1760 (*nom. cons*.). Type: *C. verum* J. S. Presl (in Berchtold & J. S. Presl, Prir. Rostlin 2: 36. 1825) (*Laurus cinnamomum* L.)

= *Cecidodaphne* Nees, Wall. Pl. Asiat. Rar. 3: 72. 1831. Type: *C. glaucescens* C. G. D. Nees.

#### 5.1.1 Diagnosis

Buds usually not perulate. Leaves usually subopposite and tripliveined, rarely alternate and pinnately veined, domatia absent, adaxial epidermal cells irregular in shape, anticlinal walls sinuous, rarely straight, periclinal walls reticulate. Inflorescences paniculate with cymes bearing strictly opposite lateral flowers. Flowers bisexual with nine fertile stamens, plus three staminodes with conspicuous cordate or sagittate heads in the fourth androecial whorl. Fruits cupulate with tepals at least partially persistent. Pedicels turbinate.

#### 5.1.2 Distribution

Tropical to subtropical Asia.

#### 5.1.3 Remarks

Several species were placed previously into sect. *Camphora* because they have seemingly pinnately veined leaves, e.g., *Cinnamomum chago*, *C. longipetiolatum*, and *C. saxatile*, but our molecular study suggests that these species belong to *Cinnamomum* (Figures 3 and 4). Similarly, Gang et al. (2021) also suggested that *C. saxatile* belongs to the former sect. *Cinnamomum* as it possesses reticulate periclinal walls. Sun and Zhao (1991) noted that leaves of *C. chago* are pinnately veined with 7–9 pairs of lateral veins, the proximal pair starting from the base of leaf blade and appearing subtriveined, suggesting that the leaf venation of the species is probably triveined. However, leaf micromorphology should be examined for these three species (and other *Cinnamomum*‐like taxa, such as *Temmodaphne thailandica*) before their taxonomic position can be confirmed. Therefore, we retain these species in *Cinnamomum* for now, pending further study.

### 5.2 Camphora

Fabr., Enum. 218. 1759. Type: *C. officinarum* Nees in Wallich, Pl. Asiat. Rar. 2: 72. 1831 ≡ *Laurus camphora* L., Sp. Pl. 1: 369. 1753

= *Parthenoxylon* Blume, Mus. Bot. 1: 322. 1851. ***Lectotype***: *Laurus parthenoxylon* W. Jack (vide Pfeiffer, Nom. 2: 598. 3 Oct 1873).

#### 5.2.1 Diagnosis

Buds usually perulate. Leaves alternate and pinnately veined or weakly tripliveined, domatia usually present in axils of lateral veins, adaxial epidermal cells polygonal, anticlinal walls straight or nearly so, periclinal walls smooth and non‐reticulate. Inflorescences paniculate with cymes bearing strictly opposite lateral flowers. Flowers bisexual with nine fertile stamens, plus three staminodes with conspicuous cordate or sagittate heads in the fourth androecial whorl. Fruits cupulate with tepals not or partially persistent. Pedicels turbinate.

#### 5.2.2 Distribution

Tropical to subtropical Asia, but mostly distributed in the Northern Hemisphere (Soh, 2011). In China, there are ca. 18 species of *Camphora*; Kochummen (1989), Soh (2011) and de Kok (2019) recorded only one species of *Camphora* (viz. *Cinnamomum porrectum* (Blume) Kosterm., synonym of *Camphora parthenoxylon* (Jack) Nees in this treatment) in the *Tree Flora of Malaya*, in Borneo and in Peninsular Malaysia and Singapore respectively; there is no species with pinnately veined leaves in southern India (Kostermans, 1985).

##### Camphora bodinieri

(H. Lév.) Y. Yang, Bing Liu & Zhi Yang, ***comb. nov*.** ≡ *Cinnamomum bodinieri* H. Lév., Repert. Spec. Nov. Regni veg. 10: 369. 1912 — ***Type***: CHINA. Guizhou (贵州, ‘Kouy‐Tchéou’): Near Guiyang (贵阳, ‘Kouy‐Yang’), “bois de la pagode de Lan‐Yo‐Chan”, 15 Jun 1899, *Bodinier 2622* (holotype: E00386445!; isotypes: P01978724 [photo!], P01978725 [photo!]; fragm. K000778561!, fragm. A00041222 [photo!], with photo of holotype).

= *Cinnamomum glanduliferum* var. *longipaniculatum* Lec., Nouv. Arch. Mus. Hist. Nat., sér. 5, 5: 74. 1913 — ***Type***: CHINA. Chongqing (重庆): Chengkou (城口, as ‘district de Tchen‐kéou‐tin’). ‘Moùng Moùng Ky’, alt. 1400 m, *Farges 894* (lectotype: P01978761!, designated here; ***isolectotypes***: K000778562!, P01964399!, P01964400!, P01964401!, P01978730!, P01978764!).

= *Cinnamomum inunctum* var. *fulvipilosum* Y.C. Yang, J. West China bord. Res. Soc. 15 (Ser. B): 73. 1945 — ***Type***: CHINA. Guizhou (贵州, as ‘Kweichow’), Zunyi (遵义), ‘Liang‐Feng‐Yah’, rocky slope near farmhouse, alt. 1000 m, 1 Aug 1931, *A. N. Steward, C. Y. Chiao* (焦启源), and H*. C. Cheo 143* (holotype: PE00028456 [photo!]; isotypes: K000778560, N102060173 [photo!], NAS00188664 [photo!], P00757069, PE00189210 [photo!], PE00028450 [photo!], PE00028457 [photo!]).

***Distribution***: Guizhou, Hubei, Hunan, Shaanxi, Sichuan, Yunnan.

##### Camphora brachythyrsa

(J. Li) Y. Yang, Bing Liu & Zhi Yang, ***comb. nov*.** ≡ *Cinnamomum brachythyrsum* J. Li, Acta Bot. Yunnan 18: 53. 1996 — ***Type***: CHINA. Yunnan (云南): Wenshan (文山), Laojunshan (老君山), May 1993, *Y.M. Shui 003072* (holotype: KUN).

***Distribution***: Yunnan.

##### Camphora chartophylla

(H.W. Li) Y. Yang, Bing Liu & Zhi Yang, ***comb. nov*.** ≡ *Cinnamomum chartophyllum* H.W. Li, acta Phytotax. Sin. 13 (4): 491975 — ***Type***: CHINA. Yunnan (云南), Menglun (勐仑), *Sheng*‐*Ji Pei* (裴盛基) *59–10384* (holotype: KUN; Isotype: fragm. L0035751)

***Distribution***: Yunnan.

##### Camphora foveolata

(Merr.) Y. Yang, Bing Liu & Zhi Yang, ***comb. nov*.** ≡ *Beilschmiedia foveolata* Merr., J. Arnold arbor. 19(1): 30. 1938 ≡ *Cinnamomum foveolatum* (Merr.) H.W.Li & J.Li, Fl. China 9: 170. 2008 ≡ *Litsea foveolata* (Merr.) Kosterm., Reinwardtia 8: 98. 1970, not yen C. Yang & P. H. Huang (1978) — ***Type***: VIETNAM. Tonkin, Chapa, alt. 1700 m, Aug., 1930, Petelot 5580 (A, BO, NY, P)

*Machilus camphorata* [“*camphoratus*”] H. Lév., Repert. Spec. Nov. Regni Veg. 9: 460. 1911 ≡ *Alseodaphne camphorata* (H.Lév.) C.K.Allen, J. Arnold Arbor. 17(4): 326 (1936) ≡ *Alseodaphne caudata* Lec. Nouv. Arch. Mus. Hist. Nat., sér. 5, 5: 97–98. 1913 ≡ *Cinnamomum caudifer* Kosterm., Reinwardtia 8: 35. 1970 — ***Type***: CHINA. Guizhou (贵州, as ‘Kouy‐Tcheou’): Guiding (贵定), Pingfa, (平伐, as ‘Pinfa’), Apr 1908 [on K000778566] or 7 May 1903 [on K000778567], *Cavalerie 1002* (holotype: E00386438; isotypes: K000778566, K000778567, L0035749, P00757048 [holotype of *Alseodaphne caudata*], fragm. A00415028).

***Distribution***: Guizhou, Yunnan; VIETNAM.

##### Camphora glandulifera

(Wall.) Nees, Pl. Asiat. Rar. 2: 72. 1831 ≡ *Laurus glandulifera* Wall., Trans. Med. Soc. Calcutta 1: 45, 51, pl. 1. 1825 ≡ *Cinnamomum glanduliferum* (Wall.) Meisn. In A. P. de Candolle, Prodr. 15 (1): 25 (1864) ≡ *Camphora rougierii* var. *glandulifera* (Nees) Lukman., Nomencl. Icon. Cannel. 23. 1889, nom. Illeg. — ***Type***: NEPAL. Mount Shivapuri [“ad Sheopore mont.”], 1821, *Wallich 2601* (holotype: K001116542; Isotypes: B100277066, B100277067, BM000880671, GZU000253941, LE00012754, Fragm. A00041270, With photo of K001116542)

= *Machilus mekongensis* Diels, Notes Roy. Bot. Gard. Edinburgh 5 (25): 244: 1912 — ***Type***: CHINA. Yunnan (云南): Weixi (维西), Cikai Town (茨开), Dong‐Shan (东山), ‘Shupa valley. Shrubby hillsides. Alt. 11,000 ft. Yangtse‐Mekong divide, Tibet’, 1904, *Forrest 370* (lectotype: E00386432, designated here).

= *Cinnamomum cavaleriei* H. Lév., Repert. Spec. Nov. Regni Veg. 10: 370. 1912 — ***Type***: CHINA. Guizhou (贵州, as ‘Kouy‐Tcheou’): Pingfa, (平伐, as ‘Pin‐Fa’), 23 Jun 1903, *Cavalerie 1084* (holotype: E00386433; isotypes: E00386434, K000778586, fragm. A00041223).

= *Machilus dominii* H. Lév., Repert. Spec. Nov. Regni Veg. 13: 174. 1914 ≡ *Cinnamomum dominii* (H. Lév.) C. F. Ji, J. Nanjing For. Univ. 25(3): 76. 2001 — ***Type***: CHINA. Yunnan (云南): Kunming (昆明), ‘Forêts de Ku‐Long‐Tchang’ (古龙场), alt. 800 m, Jul 1912, *Maire 35* (holotype: E00386435; isotypes: BM000950907, fragm. A00041227).

***Distribution***: Guizhou, Sichuan, Yunnan, Xizang; BHUTAN, INDIA, MALAYSIA, MYANMAR, NEPAL.

***Remarks***: The specimen NY00355188 labeled as isotype of *Laurus glandulifera* is not an isotype. It has been collected by *E. Meyer* in Java in 1842, and the text of Wallich was added in quotes by Meisner.

We were unable to locate the second syntype of *Machilus mekongensis*: CHINA. Yunnan (云南): Weixi (维西), Cikai Town (茨开), Dong‐Shan (东山), ‘Mekong‐Salween divide behind Tsekou mission, Tibet’, 1904, *Forrest 372*.

##### Camphora illicioides

(A. Chev.) Y. Yang, Bing Liu & Zhi Yang, ***comb. nov*.** ≡ *Cinnamomum illicioides* [“*ilicioides*”] A. Chev., bull. Écon. Indochinen. s. 20: 855. 1918 — ***Type***: VIETNAM. “Phu‐tho, Vinh‐yên, montagnes du Tam‐dao, etc.”, Phu‐tho: Trung Giáp forest reserve, 29–30 May 1918, *Fleury 37,993* (holotype: P00757044; Isotypes: K000350907, L0035795)

***Distribution***: Guangxi, Hainan; THAILAND, VIETNAM.

##### Camphora longepaniculata

(Gamble) Y. Yang, Bing Liu & Zhi Yang, ***comb. nov*.** ≡ *Cinnamomum inunctum* var. *longepaniculatum* Gamble in C. S. Sargent, Pl. Wilson. 2: 69. 1916 ≡ *Cinnamomum longepaniculatum* (Gamble) N. Chao ex H.W. Li, Acta Phytotax. Sin. 13 (4): 48, f. 2. 1975 — ***Type***: CHINA. Sichuan (四川): Ya'an (雅安, as ‘Yachou Fu’), 600–1000 m, Jun 1980, *Wilson 3710* (holotype: A00041232; Isotypes: BM000950908, HBG509751, US00099081)

***Distribution***: Sichuan.

##### Camphora micrantha

(Hayata) Y. Yang, Bing Liu & Zhi Yang, ***comb. nov*.** ≡ *Machilus micrantha* Hayata, icon. Pl. Formosan. 2: 130. 1912 ≡ *Cinnamomum micranthum* (Hayata) Hayata, Icon. Pl. Formosan. 3: 160. 1913 — ***Type***: CHINA. Taiwan (台湾): Xinbei City (新北市), Sanxia (插角[三峡], 大豹), as “Sankakuyu, Taihyo”, *Kanehira s.n*., Jun 1912 (*holotype*: TI No. 02537)

= *Cinnamomum kanehirae* [“*kanehirai*”] Hayata, Icon. Pl. Formosan. 3: 159. 1913 ≡ *C. micranthum* f. *kanehirai* (Hayata) S. S. Ying, Mém. Coll. Agric. Natl. Taiwan Univ. 25 (1): 108. 1986 — ***Type***: CHINA. Taiwan (台湾): Miaoli (苗栗), Nanzhuang (南庄, 加里前山), as “Nanshoshicho, Kalizenzan”, alt. 4000 ft., Oct. 1912, *Kanehira s.n*. (holotype: TI2456).

= *Cinnamomum xanthophyllum* H. W. Li, Acta Phytotax. Sin. 13(4): 47. 1975 — ***Type***: CHINA. Guangdong (广东): Xinfeng (新丰), alt. 650 m, *L. Deng* (邓良) *8043* (holotype: KUN; isotypes: IBK00004390, IBSC0046454, SZ00162655, AU034001, PE00189952).

***Distribution***: Fujian, Guangdong, Guangxi, Guizhou, Hainan, Jiangxi, Taiwan; VIETNAM.

##### Camphora migao

(H.W. Li) Y. Yang, Bing Liu & Zhi Yang, ***comb. nov*.** ≡ *Cinnamomum migao* H.W. Li, Acta Phytotax. Sin. 16 (2): 90, pl. 7, f. 1. 1978 — ***Type***: CHINA. Yunnan (云南): Funing (富宁), alt. 500 m, *H. T. Tsai* (蔡希陶) *58–9048* (holotype: KUN; Isotypes: IBK00200070, LBG00072386)

***Distribution***: Guangxi, Yunnan.

##### Camphora mollifolia

(H.W. Li) Y. Yang, Bing Liu & Zhi Yang, ***comb. nov*.** ≡ *Cinnamomum mollifolium* H.W. Li, Acta Phytotax. Sin. 13 (4): 45, f. 1. 1975 — ***Type***: CHINA. Yunnan (云南): Menghai (勐海), *Y. H. Li* (李延辉) *60–11,664* (***holotype***: KUN48456; Isotype: Fragm. L0035915)

***Distribution***: Yunnan.

##### Camphora officinarum

Nees, Pl. Asiat. Rar. 2: 721831 ≡ *Laurus camphora* L., Sp. Pl. 1: 369. 1753 ≡ *Persea camphora* (L.) Spreng., Syst. Veg.2: 268. 1825 ≡ *Cinnamomum camphora* (L.) J. Presl, Prir. Rostlin 2: 36, pl. 8. 1825 ≡ *Camphora officinalis* Steud., Nomencl. Bot., ed. 2 (Steudel) 1: 271. 1840 ≡ *Camphora camphora* (L.) H. karst., Deut. Fl. 504. 1881, *nom. inval*. — ***Type***: **Japan**. Locality and date not indicated, *collector not indicated* (lectotype: LINN 518.7 [photo!], designated by Kostermans, 1978)

= *Camphora japonica* Garsault, Descr. Vert. Usag. 719. Pl. 1: 61, t. 84. 1767 — ***Holotype***: **Japan**. no collection indicated; plate 84 may be accepted as type.

= *Camphora officinarum* var. *glaucescens* A. Braun, Verh. Vereins Beförd. Gartenbaues Königl. Preuss. Staaten 21: 77 (1853); *Cinnamomum camphora* var. *glaucescens* (A. Braun) Meisn. in A. P. de Candolle, Prodr. 15 (1): 24. 1864 — ***Type***: cultivated, presumably in Berlin (***holotype***: B, possibly destroyed; ***isotype***: P01991848).

= *Cinnamomum camphora* fo. *parvifolia* Miq., Ann. Mus. Bot. Lugduno‐Batavi 2: 195. 1866 — ***Type***: **Japan**. Nagasaki: 1862–1863, *Oldham 704* (holotype: L0308679 fruiting with Miquel's handwriting; isotype P00757059, probable type of *Camphora humboldtii*, see below).

= *Camphora oldhamii* Lukman., Nomencl. Icon. Cannel. 23. 1889 — ***probable type*** (fide Kostermans): CHINA. Taiwan (台湾): 1864, *Oldham 44?* [interpreted as *4425* in P; could equally well be *447* or *449*] (P00757057).

= *Cinnamomum camphora* var. *nominale* Hayata, J. Coll. Sci. Imp. Univ. Tokyo 22: 349. 1906 ≡ *C. nominale* (Hayata) Hayata, Icon. Pl. Formosan. 3: 160. 1913 — ***Holotype***: CHINA. Taiwan (台湾): Hengchun [“Koshun”], 1905, *Kawakami* (not found); ***neotype*** (designated here): CHINA. Taiwan (台湾): Pingdong (屏东), Ken‐Ding‐Park (垦丁公园), Kuraru (龟子角, as ‘Koshun’), May 30th, 1912, *B. Hayata s.n*. (TI no. 02459 [photo!]; ***isoneotypes***: TI nos. 02460 [photo!], 02461 [photo!], 02462 [photo!]).

= *Cinnamomum taquetii* H. Lévl., Feddes Repert. 10: 370. 1912 — Type: SOUTH KOREA. Jeju Island (济州岛, as ‘Quelpaert’), Daejeong‐eup (大静, ‘in silvis Taitpjeng’), Jul 1909, *Taquet 3159* (Lectotype: E00386436 [photo!], designa; isolectotypes: KYO, TI).

= *Cinnamomum camphoroides* Hayata, Icon. Pl. Formosan. 3: 158. 1913 — ***Type***: CHINA. Taiwan (台湾): Pingdong (屏东), Koshun (恒春), *N.Konishi s.n*. (holotype: TI 2048; isotype: L0035885).

= *Cinnamomum nominale* var. *lanata* Nakai, Fl. Sylv. Kor. 22: 301939 — ***Typ*** e: CHINA. Taiwan (台湾): Hualiangang (花莲港, as ‘Kwarenko’), date and collector not indicated (holotype: TI no. 02463).

= *Cinnamomum simondii* Lecomte, J. Arnold Arbor. 20: 45 (1939). — ***Type***: CHINA. Guangxi (广西): Longzhou (龙州, as ‘Long Tchéou’), without date, *Simond 190* (holotype: P00757021; ***isotypes***: K000778667, L0035964; photo of holotype, A00041278).

= *Cinnamomum camphora* var. *cyclophyllum* Nakai, J. Jap. Bot. 19: 369. 1943 — ***Type***: SOUTH KOREA. Jeju Island (济州岛, as ‘Saisyuto’), ‘in sylvis montis Kanrasan’ (汉拏山), 10 Nov. 1917, *Takahasi s.n*. (holotype: TI no. 02444).

= *Cinnamomum camphora* var. *linaloolifera* Y. Fujita, Bot. Mag. (Tokyo) 65: 2451952 ≡ *Cinnamomum camphora* f. *linaloolifera* (Y. Fujita) Sugim., New Keys Jap. Trees 459. 1961 — ***Type***: CHINA. Taiwan (台湾): ‘Hab. Formosa’, collector and date not indicated (not seen).

***Distribution***: wide spread in southern China; Japan, Korea, Vietnam.

##### Camphora parthenoxylon

(Jack) Nees in wall. Pl. Asiat. Rar. 2: 72. 1831 ≡ *Laurus parthenoxylon* Jack, Malayan Misc. 1(5): 28. 1820 ≡ *Camphora parthenoxylon* (Jack) Nees in Wall., Pl. Asiat. Rar. 2: 72. 1831 ≡ s*assafras parthenoxylon* (Jack) Nees, syst. Laur. 491. 1836 ≡ *Cinnamomum parthenoxylon* (Jack) Meisn. In A. Candolle Prodr. 15(1): 26. 1864 — ***Type***: [INDONESIA]. Sumatra, ‘*kayo Gadis*’, *herb. Roxburgh s.n*. (holotype: BR0000005931088; Isotype: BR0000005931132)

≡ *Laurus porrecta* Roxb., Hort. Beng. 30. 1814, nom. Inval. ≡ *Camphora porrecta* (Roxb.) Voigt, Hort. Suburb. Calc. 308. 1845, *nom*. inval. ≡ *Parthenoxylon porrectum* Blume, Mus. Bot. 1: 323. 1851 ≡ *Cinnamomum porrectum* (Blume) Kosterm. J. Sci. Res. (Jakarta) 1(5): 126. 1952 — ***Type***: [INDONESIA. Sumatra or India, cult. Hort. Bot. Calcutta] *Roxburgh s.n*. (neotype: BR0000005931088, designated by Kostermans, 1970, second‐step by Turner, 2013; isoneotype: BR0000005931132).

= *Cinnamomum barbatoaxillatum* N. Chao in Fl. Sichuan. 1: 36, 459. 1981 — ***Type***: CHINA. Sichuan (四川): Yibin (宜宾), fruit, *N. Zhao 2918* (holotype: SCFI; isotypes: IBK00345678, KUN0101453).

***Distribution***: Fujian, Guangdong, Guangxi, Guizhou, Hainan, Hunan, Jiangxi, Sichuan, Yunnan; BHUTAN, CAMBODIA, INDIA, INDONESIA, LAOS, MALAYSIA, MYANMAR, NEPAL, PAKISTAN, THAILAND, VIETNAM.

##### Camphora philippinensis

(Merr.) Y. Yang, Bing Liu & Zhi Yang, ***comb. nov*.** ≡ *Machilus philippinensis* Merr., Philipp. J. Sci. 1 (suppl. 1): 561906 ≡ *Persea philippinensis* (Merr.) Elmer, Leafl. Philipp. Bot. 2: 384. 1908 ≡ *Cinnamomum philippinense* (Merr.) C.E. Chang, Fl. Taiwan 2: 417. 1976 — ***Type***: PHILIPPINES. Luzon: Prov. Bataan, Lamao River, mt. Mariveles, mar 1905, *Meyer 2793* (***lectotype***: US00516627; ***Isolectotypes***: NY00355328, NY00355329, NY00355330)

= *Cinnamomum acuminatissimum* Hayata, Icon. Pl. Formosan. 3: 157–158. 1913 ≡ *Machilus acuminatissima* (Hayata) Kaneh., Formosan Trees (rev. ed.) 219. 1936 ≡ *Persea acuminatissima* (Hayata) Kosterm., Reinwardtia 6 (2): 191. 1962 — ***Type***: CHINA. Taiwan (台湾): Hualian (花莲), Taisho (大庄), March 26th, 1911, *Furukawa s.n*. (***holotype***: TI no. 02442; possible ***isotypes***: K000778559, fragm. L0035683).

= *Cinnamomum caudatifolium* Hayata, Icon. Pl. Formosan. 5: 155, f. 54b. 1915 — ***Type***: CHINA. Taiwan (台湾): Jiayi (嘉义县), Alishan [“Mt. Arisan”], between Karapin (Chaoliping交力坪) and Fenchihu [奋起湖, as “Funkiko”], near Shuisheliao [水车竂, as “Suisharyo”], [27] Mar. 1914, *Hayata s.n*. (***holotype***: TI no. 02450; ***isotype***: fragm. L0035684).

***Distribution***: Taiwan; PHILIPPINES.

***Remarks***: We selected the collection *Meyer 2793* (US00516627) as lectotype of *Machilus philippinensis* because it bears an original data sheet and apparently has been annotated by Merrill. The two other syntypes are: PHILIPPINES. Luzon: Prov. Bataan, Lamao River, Mt. Mariveles, Mar 1905, *Whitford 1139* (K000778822, NY00355330, US00099164); PHILIPPINES. Luzon: Prov. Bataan, Lamao River, Mt. Mariveles, Apr 1905, *Whitford 1220* (K000778823, NY00355328, US00516628). The collection *Elmer 8184*, labeled in several herbaria as type, has been cited by Elmer, but is not a type.

##### Camphora platyphylla

(Diels) Y. Yang, Bing Liu & Zhi Yang, ***comb. nov*.** ≡ *Machilus platyphylla* Diels, Bot. Jahrb. Syst. 29: 348. 1900 ≡ *Cinnamomum platyphyllum* (Diels) Allen, J. Arnold arbor. 20: 46. 1939 — ***Lectotype*** (designated here): CHINA. Chongqing (重庆): Nanchuan (南川), *native collectors, commun. Bock & von Rosthorn 1981* (A00041247; isolectotype: fragm. L0035914)

= *Cinnamomum chengkouense* N. Chao, Fl. Sichuan. 1: 459*–*460, f. 13. 1981 —***Type***: CHINA. Chongqing (重庆): Chengkou (城口), *T. L. Dai* (戴天伦) *102,187* (holotype: SZ; isotypes: PE00189181, IBK00190147).

***Distribution***: Chongqing, Sichuan.

##### Camphora purpurea

(H.G. Ye & F.G. Wang) Y. Yang, Bing Liu & Zhi Yang, ***comb. nov*.** ≡ *Cinnamomum purpureum* H.G. Ye & F.G. Wang, Novon, 16: 439. 2006. — ***Holotype***: CHINA. Guangdong (广东): Yangchun city (阳春市), Ehuangzhang mtn., safflower pond, ca. 300–800 m, 2 mar. 2002, *ye Hua*‐*gu & ye Yu*‐*shi 6892* (IBSC; isotypes, IBSC)

***Distribution***: Guangdong.

***Remarks***: This name was treated as a synonym of *Cinnamomum parthenoxylon* (Jack) Meisn. in the *Flora of China* (Li et al., 2008). However, this species markedly differs from the latter in the purplish color of its branchlets, petioles, pedicel, and peduncles. We thus treat it as a separate species here.

##### Camphora rufotomentosa

**(**K.M. Lan) Y. Yang, Bing Liu & Zhi Yang, ***comb. nov*.** ≡ *Cinnamomum rufotomentosum* K.M. Lan, Fl. Guizhou 2: 674. Pl. 32. 1984 — Holotype: CHINA. Guizhou (贵州): Xingyi (兴义), *K.M. Lan 40* (GZAC).

***Distribution***: Guizhou.

##### Camphora septentrionalis

(Hand.‐Mazz.) Y. Yang, Bing Liu & Zhi Yang, ***comb. nov*.** ≡ *Cinnamomum septentrionale* Hand.‐Mazz., Oesterr. Bot. Z. 85: 213–214. 1936 — ***Type***: CHINA. Shaanxi (陕西): N side of tapa‐Shan near Hanzhong (汉中, as ‘Hantschung’), Xiao‐Nan‐Hai, 800 m, May‐Jun 1934, *Fenzel 633* (holotype: W, probably destroyed in world war II; isotypes: fragm. A00246778, with photo of holotype [left image]; L0035916; PE00294191)

= *Cinnamomum inunctum* var. *albosericeum* Gamble in C. S. Sargent, Pl. Wilson. 2: 69. 1916 — ***Type***: CHINA. Sichuan (四川): Mianzhu Xian [as “Mien‐chu Hsien”, 绵竹县], 600 m, 19 May 1908, *Wilson 3713* (holotype: A00041231; isotypes: B100277110, HBG509750, HUHA00041231 [photo!], IBSC0046892, IBSC0046891, L0035712, US00099080).

***Distribution***: Gansu, Shaanxi, Sichuan.

##### Camphora tenuipilis

(Kosterm.) Y. Yang, Bing Liu & Zhi Yang, ***comb. nov*.** ≡ *Alseodaphne mollis* W.W. Sm., notes Roy. Bot. Gard. Edinburgh 13: 153–154. 1921; ≡ *Cinnamomum tenuipile* (“*tenuipilis*”) Kosterm., Reinwardtia 8: 74. 1970 — ***Type*** : CHINA. Yunnan (云南): Shweli‐Salween divide, in thickets. Lat. 25°30′N. Alt. 9000 ft., oct 1917, *Forrest 16,021* (lectotype: E00123606, designated by Kostermans, 1970; isolectotype: K000350906).

***Distribution***: Yunnan.

***Remarks***: *Alseodaphne mollis* was based on two syntypes. The other syntype is: CHINA. Yunnan: Salween Valley, in open thickets. Lat. 25°6′ N. Alt. 4000 ft., Apr. 1917, *Forrest 13,667* (E00123605; isosyntype: K000350905). When Kostermans (1970) transferred the species to *Cinnamomum*, he wrote “Typus: *Forrest 16021* (E), syntypus: *Forrest 13667* (E).” This may be interpreted as lectotypification in the sense of the fruiting specimen E00123606, even though E00123605 is the better flowering material.

## AUTHOR CONTRIBUTIONS

**Zhi Yang:** Data curation (lead); formal analysis (lead); methodology (lead); software (lead); writing – original draft (equal). **Bing Liu:** Conceptualization (supporting); investigation (supporting); resources (lead); visualization (lead); writing – review and editing (equal). **Yong Yang:** Conceptualization (lead); funding acquisition (lead); investigation (lead); project administration (lead); supervision (lead); writing – original draft (lead); writing – review and editing (lead). **David Kay Ferguson:** Investigation (supporting); writing – review and editing (supporting).

## CONFLICT OF INTEREST

The authors declare that there is no conflict of interest.

## Data Availability

All data used in the study are included in this paper.

## APPENDIX A Sequences obtained from GenBank for phylogenetic analysis

**Table:**

Taxon	nrITS	*psb*A‐*trn*H	Plastome	Clade
*Aiouea acarodomatifera*	MF110006.1	–	–	I
*Aiouea alainii*	MF110007.1	–	–	I
*Aiouea amoena*	MF110008.1	MF137928.1	–	I
*Aiouea chavarriana*	MF110009.1	MF137929.1	–	I
*Aiouea cinnamomoidea*	AF272288.1	–	–	I
*Aiouea dubia*	MF110012.1	MF137932.1	–	I
*Aiouea erythropus*	AF272264.1	–	–	I
*Aiouea formicaria*	KX509823.2	KX509883.1	–	I
*Aiouea grandifolia*	MF110014.1	MF137934.1	–	I
*Aiouea guianensis*	AF272251.1	AF268780.1	–	I
*Aiouea hammeliana*	MF110016.1	MF137936.1	–	I
*Aiouea haussknechtii*	MF110019.1	MF137937.1	–	I
*Aiouea hirsuta*	KX509824.1	KX509884.1	–	I
*Aiouea maya*	MF110020.1	MF137940.1	–	I
*Aiouea montana*	MF110021.1	MF137941.1	–	I
*Aiouea myristicoides*	MF110022.1	MF137942.1	–	I
*Aiouea obscura*	MK507230.1	MK507298.1	–	I
*Aiouea padiformis*	KU139868.1	KU160284.1	–	I
*Aiouea palaciosii*	MF110023.1	MF137943.1	–	I
*Aiouea pittieri*	KU139836.1	–	–	I
*Aiouea saligna*	KX509821.1	KX509881.1	–	I
*Aiouea sellowiana*	MF110025.1	MF137945.1	–	I
*Aiouea subsessilis*	KU139889.1	KU160287.1	–	I
*Aiouea tetragona*	AF272265.1	AF268781.1	–	I
*Aiouea tomentosa*	MF110031.1	MF137951.1	–	I
*Aiouea tomentosa*	FM957804.1	–	–	I
*Aiouea trinervis*	MF110032.1	MF137952.1	–	I
*Alseodaphne semecarpifolia*	MG188583.1	–	NC_037491.1	Outgroup
*Aniba affinis*	MK507231.1	MK507299.1	–	I
*Aniba canelilla*	MW489499.1	–	–	I
*Aniba excelsa*	AF272255.1	–	–	I
*Aniba firmula*	MF110034.1	MF137954.1	–	I
*Aniba heringeri*	GQ480364.1	–	–	I
*Aniba panurensis*	AF272256.1	GQ428739.1	–	I
*Aniba parviflora*	MW489500.1	KX248005.1	–	I
*Aniba rosaeodora*	MW489501.1	MT679556.1	–	I
*Aniba taubertiana*	MK507233.1	KX248016.1	–	I
*Aniba terminalis*	MW489502.1	KX248008.1	–	I
*Cinnamomum agasthyamalayanum*	MH232437.1	–	–	IV
*Cinnamomum appelianum*	KP092853.1	KX546093.1	–	IV
*Cinnamomum aromaticum*		–	NC_046019.1	IV
*Cinnamomum austrosinense*	KU139818.1	KJ686727.1	–	IV
*Cinnamomum austroyunnanense*	KU139819.1	–	–	IV
*Cinnamomum baileyanum*	KU139820.1	KU160274.1	–	IV
*Cinnamomum bejolghota*	KX546412.1	EU153949.1	–	IV
*Cinnamomum bodinieri*	MH270478.1	MF137955.1	–	II
*Cinnamomum burmanni*	MF110036.1	MF137958.1	MW421305.1	IV
*Cinnamomum camphora*	KT248576.1	GU135428.2	MF421523.1	II
*Cinnamomum celebicum*	KU139829.1	–	–	IV
*Cinnamomum chago*	KU139830.1	KX546108.1	MN047449.1	IV
*Cinnamomum chartophyllum*	KU139832.1	–	MW421301.1	II
*Cinnamomum chemungianum*	MH232440.1	–	–	IV
*Cinnamomum cordatum*	KU139835.1	–	–	IV
*Cinnamomum crenulicupulum*	KU139838.1	–	–	IV
*Cinnamomum curvifolium*	KU139895.1	HM019397.1	–	IV
*Cinnamomum cuspidatum*	KU139839.1	–	–	IV
*Cinnamomum daphnoides*	MF110043.1	MF137962.1	–	IV
*Cinnamomum doederleinii*	KU139842.1	–	–	IV
*Cinnamomum dubium*	MH232442.1	MW408304.1	–	IV
*Cinnamomum filipedicellatum*	MH232443.1	–	–	IV
*Cinnamomum foveolatum*	MT628595.1	–	–	II
*Cinnamomum glanduliferum*	KX546415.1	MF137966.1	NC_057217.1	II
*Cinnamomum goaense*	MH232446.1	–	–	IV
*Cinnamomum heyneanum*	KU139847.1	MG209136.1	SY9391	IV
*Cinnamomum insularimontanum*	KY271510.1	AF268782.1	OL544942.1	IV
*Cinnamomum javanicum*	KU139852.1	–	–	IV
*Cinnamomum jensenianum*	KU139853.1	HM019391.1	–	IV
*Cinnamomum kalbaricum*	KU139844.1	–	–	IV
*Cinnamomum keralaense*	MH232450.1	–	–	IV
*Cinnamomum kotoense*	KY271518.1	KY296429.1	NC_050346.1	IV
*Cinnamomum laubatii*	KU139855.1	–	–	IV
*Cinnamomum liangii*	KU139856.1	–	–	IV
*Cinnamomum litseifolium*	MH232451.1	MW408307.1	–	IV
*Cinnamomum longipaniculatum*	KX546420.1	KY223702.1	MW553040.1	II
*Cinnamomum longipetiolatum*	KU139858.1	–	–	IV
*Cinnamomum loureiroi*	MF110051.1	MF137971.1	–	IV
*Cinnamomum macrocarpum*	MN519276.1	–	–	IV
*Cinnamomum mairei*	KU139859.1	–	–	IV
*Cinnamomum malabatrum*	MH232453.1	KY966336.1	–	IV
*Cinnamomum micranthum*	KU139860.1	KJ686734.1	NC_035802.1	II
*Cinnamomum migao*		–	NC_058709.1	II
*Cinnamomum mohananianum*	MH232466.1	–	–	IV
*Cinnamomum mollifolium*	KU139861.1	–	MW421302.1	II
*Cinnamomum nilagiricum*	MH232469.1	–	–	IV
*Cinnamomum okinawense*	KU139863.1	–	–	IV
*Cinnamomum oliveri*	KU139865.1	–	KT716496.1	IV
*Cinnamomum osmophloeum*	KY271528.1	KY296439.1	MT384386.1	IV
*Cinnamomum paiei*	MF110053.1	MF137973.1	–	IV
*Cinnamomum parthenoxylon*	KX546421.1	KX546120.1	MH050971.1	II
*Cinnamomum pauciflorum*	–	–	MW421303.1	IV
*Cinnamomum perrottetii*	MH232470.1	–	–	IV
*Cinnamomum pingbienense*	KU139873.1	–	–	IV
*Cinnamomum pittosporoides*	DQ124269.1	KX546125.1	NC_048978.1	IV
*Cinnamomum platyphyllum*	KU139875.1	HM019396.1	–	II
*Cinnamomum polderi*	MF110056.1	MF137976.1	–	IV
*Cinnamomum propinquum*	KU139876.1	–	–	IV
*Cinnamomum racemosum*	MF110044.1	MF137964.1	–	IV
*Cinnamomum reticulatum*	KU139879.1	MF623431.1	–	IV
*Cinnamomum rhynchophyllum*	KU139880.1	–	–	IV
*Cinnamomum rigidissimum*	KU139881.1	–	–	IV
*Cinnamomum riparium*	MH232473.1	MF072390.1	–	IV
*Cinnamomum saxatile*	KU139882.1	–	–	IV
*Cinnamomum septentrionale*	KU139883.1	–	MW421306.1	II
*Cinnamomum sintoc*	KU139884.1	–	–	IV
*Cinnamomum subavenium*	GU598528.1	KJ686740.1	MW801140.1	IV
*Cinnamomum tamala*	MF110058.1	MH232535.1	–	IV
*Cinnamomum tavoyanum*	MH232475.1	MF072387.1	–	IV
*Cinnamomum tazia*	KP092859.1	KX546123.1	–	IV
*Cinnamomum tenuifolium*	KU139892.1	HQ427110.1	–	II
*Cinnamomum tenuipile*	KU139893.1	KR533062.1	NC_057069.1	IV
*Cinnamomum travancoricum*	MH232479.1	MF547522.1	–	IV
*Cinnamomum tsangii*	KU139900.1	–	–	IV
*Cinnamomum tsoi*	KU139901.1	–	–	IV
*Cinnamomum verum*	KU139902.1	MH232541.1	NC_035236.1	IV
*Cinnamomum walaiwarense*	MH232490.1	–	–	IV
*Cinnamomum wightii*	MH232487.1	–	–	IV
*Cinnamomum wilsonii*	KU139904.1	KY223672.1	MW800949.1	IV
*Cinnamomum yabunikkei*	–	–	NC_044864.1	IV
*Damburneya ambigens*	KX509828.1	KX509888.1	–	I
*Damburneya colorata*	MK507234.1	MK507302.1	–	I
*Damburneya coriacea*	MF110063.1	MF137983.1	–	I
*Damburneya gentlei*	KX509830.1	KX509890.1	–	I
*Damburneya guatemalensis*	MF110015.1	MF137935.1	–	I
*Damburneya inconspicua*	MK507235.1	MK507303.1	–	I
*Damburneya martinicensis*	KX509831.1	KX509891.1	–	I
*Damburneya minima*	MK507236.1	MK507304.1	–	I
*Damburneya parvissima*	MK507237.1	MK507305.1	–	I
*Damburneya patens*	KX509832.1	KX509892.1	–	I
*Damburneya purpurea*	AF272293.1	EU153974.1	–	I
*Damburneya salicifolia*	AF272294.1	–	–	I
*Damburneya smithii*	MK507238.1	MK507306.1	–	I
*Damburneya umbrosa*	MK507239.1	MK507307.1	–	I
*Dicypellium caryophyllaceum*	MK507240.1	–	–	I
*Dicypellium manausense*	AF272270.1	AF268775.1	–	I
*Endlicheria anomala*	AF363371.1	–	–	I
*Endlicheria bracteolata*	AF363372.1	–	–	I
*Endlicheria chalisea*	MK507241.1	AF268756.1	–	I
*Endlicheria citriodora*	MK507242.1	AF268757.1	–	I
*Endlicheria dysodantha*	AF363373.1	–	–	I
*Endlicheria glomerata*	MF110064.1	MF137984.1	–	I
*Endlicheria gracilis*	AF363374.1	–	–	I
*Endlicheria longicaudata*	AF363375.1	MK507311.1	–	I
*Endlicheria metallica*	AF363376.1	–	–	I
*Endlicheria multiflora*	AF363377.1	MF786039.1	–	I
*Endlicheria paniculata*	AF363378.1	–	–	I
*Endlicheria pyriformis*	MF110066.1	MF137986.1	–	I
*Endlicheria reflectens*	AF272274.1	AF268758.1	–	I
*Endlicheria rubra*	AF363380.1	–	–	I
*Endlicheria ruforamula*	AF363381.1	–	–	I
*Endlicheria sprucei*	AF363382.1	–	–	I
*Endlicheria szyszylowiczii*	MF110067.1	MF137987.1	–	I
*Endlicheria tessmannii*	AF363383.1	–	–	I
*Kubitzkia mezii*	AF272276.1	AF268772.1	–	I
*Kuloa ikonyokpe*	AF272305.1	–	–	V
*Kuloa usambarensis*	MN431689.1	MN431714.1	–	V
*Licaria armeniaca*	MK507245.1	MK507314.1	–	I
*Licaria bahiana*	MF110068.1	MF137988.1	–	I
*Licaria cannella*	AF272280.1	AF268773.1	–	I
*Licaria crassifolia*	MF110069.1	MF137989.1	–	I
*Licaria guianensis*	AF272281.1	KX248791.1	–	I
*Licaria martiniana*	AF272279.1	KX248795.1	–	I
*Licaria pachycarpa*	MK507247.1	MK507316.1	–	I
*Licaria rodriguesii*	MK507248.1	MK507317.1	–	I
*Licaria triandra*	AF272282.1	AF268774.1	–	I
*Machilus thunbergii*	KX546512.1	–	NC_038204.1	Outgroup
*Mespilodaphne cymbarum*	MK507249.1	MK507318.1	–	I
*Mespilodaphne quixos*	MF110080.1	KX509937.1	OM135246.1	I
*Mespilodaphne veraguensis*	AF272319.1	–	–	I
*Nectandra acutifolia*	KX509834.1	KX509894.1	–	I
*Nectandra amazonum*	FM957816.1	KX509895.1	–	I
*Nectandra angusta*	KX509835.1	KX509896.1	–	I
*Nectandra angustifolia*	KF420965.1	KF421030.1	MF939340.1	I
*Nectandra apiculata*	KX509836.1	KX509897.1	–	I
*Nectandra barbellata*	KX509837.1	KX509898.1	–	I
*Nectandra citrifolia*	KX509842.1	–	–	I
*Nectandra cuneatocordata*	KX509843.1	KX509903.1	–	I
*Nectandra cuspidata*	AF272291.1	EU153966.1	–	I
*Nectandra discolor*	KX509844.1	KX509904.1	–	I
*Nectandra grandiflora*	KF420973.1	KF421022.1	–	I
*Nectandra herrerae*	KX509846.1	KX509906.1	–	I
*Nectandra hihua*	KX509847.1	KJ426843.1	–	I
*Nectandra lanceolata*	KF420966.1	KF421026.1	–	I
*Nectandra latissima*	KX509848.1	KX509909.1	–	I
*Nectandra laurel*	KX509849.1	KX509910.1	–	I
*Nectandra lineata*	KX509839.1	EU153970.1	–	I
*Nectandra lineatifolia*	KX509851.1	KX509912.1	–	I
*Nectandra matthewsii*	KX509840.1	KX509900.1	–	I
*Nectandra maynensis*	KX509853.1	KX509914.1	–	I
*Nectandra micranthera*	KX509855.1	KX509916.1	–	I
*Nectandra microcarpa*	KX509856.1	KX509917.1	–	I
*Nectandra nitidula*	KX509857.1	KX509918.1	–	I
*Nectandra obtusata*	KX509858.1	KX509919.1	–	I
*Nectandra olida*	KX509859.1	KX509920.1	–	I
*Nectandra oppositifolia*	KX509860.1	KX509921.1	–	I
*Nectandra paranaensis*	KX509861.1	KX509922.1	–	I
*Nectandra paucinervia*	KX509862.1	KX509923.1	–	I
*Nectandra psammophila*	MF110070.1	KX509924.1	–	I
*Nectandra puberula*	KX509863.1	KX509925.1	–	I
*Nectandra pulverulenta*	KX509864.1	KX509926.1	–	I
*Nectandra reflexa*	KX509865.1	KX509927.1	–	I
*Nectandra turbacensis*	AF272295.1	AF268768.1	–	I
*Nectandra villosa*	GQ480373.1	KX509928.1	–	I
*Ocotea aciphylla*	DQ787422.1	MF137994.1	–	I
*Ocotea ambrensis*	MN431690.1	MN431716.1	–	I
*Ocotea arcuata*	MK507250.1	MK507319.1	–	I
*Ocotea atirrensis*	MF110071.1	MF137995.1	–	I
*Ocotea aurantiodora*	MK507251.1	MK507320.1	–	I
*Ocotea auriculiformis*	MN431691.1	MN431717.1	–	I
*Ocotea balanocarpa*	MK507252.1	MK507321.1	–	I
*Ocotea bicolor*	GQ480375.1	–	–	I
*Ocotea botrantha*	KX509867.1	KX509930.1	–	I
*Ocotea brachybotrya*	GQ480376.1	–	–	I
*Ocotea brenesii*	MK507253.1	MK507322.1	–	I
*Ocotea bullata*	AF272298.1	AF268778.1	–	I
*Ocotea caniflora*	MK507254.1	MK507323.1	–	I
*Ocotea catharinensis*	KF420963.1	KF421033.1	–	I
*Ocotea ceanothifolia*	AF272299.1	KX248927.1	–	I
*Ocotea ciliata*	MF110072.1	MF137996.1	–	I
*Ocotea comoriensis*	MN431692.1	MN431718.1	–	I
*Ocotea complicata*	MK507256.1	MK507325.1	–	I
*Ocotea congregata*	MK507257.1	MK507326.1	–	I
*Ocotea corymbosa*	GQ480377.1	–	–	I
*Ocotea cujumary*	MK507258.1	MK507327.1	–	I
*Ocotea cymosa*	MN431693.1	MN431719.1	–	I
*Ocotea daphnifolia*	MK507259.1	MK507328.1	OM135247.1	I
*Ocotea dentata*	MK507260.1	MK507329.1	–	I
*Ocotea diospyrifolia*	GQ480379.1	–	–	I
*Ocotea divaricata*	MK507261.1	MK507330.1	–	I
*Ocotea domatiata*	MK507262.1	MK507331.1	–	I
*Ocotea elegans*	MF110073.1	MF137997.1	–	I
*Ocotea fasciculata*	MK507263.1	MK507332.1	–	I
*Ocotea floribunda*	KX509868.1	KX509931.1	–	I
*Ocotea foetens*	AF272300.1	MN431720.1	OM135248.1	I
*Ocotea gabonensis*	MF110075.1	MF138000.1	–	I
*Ocotea glaucosericea*	MK507264.1	MK507333.1	–	I
*Ocotea glomerata*	GQ480380.1	–	–	I
*Ocotea grayi*	MN431697.1	MN431724.1	–	I
*Ocotea guatemalensis*	MK507266.1	MK507335.1	–	I
*Ocotea guianensis*	AF272302.1	AF268761.1	OM135249.1	I
*Ocotea heydeana*	AF272304.1	–	–	I
*Ocotea holdridgeana*	MK507267.1	MK507337.1	–	I
*Ocotea indecora*	MF110076.1	MF138001.1	–	I
*Ocotea insularis*	MK507269.1	MK507339.1	–	I
*Ocotea involuta*	MN431698.1	MN431725.1	–	I
*Ocotea javitensis*	MK507270.1	MK507340.1	–	I
*Ocotea kenyensis*	MN114140.1	MN431726.1	–	I
*Ocotea keriana*	MK507271.1	MK507341.1	–	I
*Ocotea laetevirens*	MK507272.1	MK507342.1	–	I
*Ocotea lancifolia*	GQ480383.1	–	–	I
*Ocotea laxa*	MK507273.1	MK507343.1	–	I
*Ocotea lentii*	MK507274.1	MK507344.1	–	I
*Ocotea leptobotra*	MK507275.1	–	–	I
*Ocotea leucoxylon*	KX509852.1	KX509913.1	–	I
*Ocotea longifolia*	GQ480385.1	–	–	I
*Ocotea longipes*	MN431701.1	MN431728.1	–	I
*Ocotea macrocarpa*	MN431702.1	MN431729.1	–	I
*Ocotea macrophylla*	AF272303.1	MK507336.1	–	I
*Ocotea magnilimba*	KX509870.1	KX509932.1	–	I
*Ocotea malcomberi*	AF272307.1	AF268779.1	–	I
*Ocotea mascarena*	MN431703.1	MN431730.1	–	I
*Ocotea meziana*	MK507276.1	MK507345.1	–	I
*Ocotea micans*	MK507277.1	MK507346.1	–	I
*Ocotea minarum*	MK507278.1	MK507347.1	–	I
*Ocotea montana*	MK507279.1	MK507348.1	–	I
*Ocotea nervosa*	MN431704.1	MN431731.1	–	I
*Ocotea nigra*	AF272308.1	KX248946.1	–	I
*Ocotea nitida*	GQ480387.1	MK507349.1	–	I
*Ocotea oblonga*	MK507280.1	EU153984.1	–	I
*Ocotea odorifera*	KX509871.1	KF421038.1	OM135250.1	I
*Ocotea pauciflora*	MK507281.1	AF268764.1	–	I
*Ocotea percoriacea*	AF272311.1	MK507351.1	–	I
*Ocotea perforata*	MN431705.1	MN431732.1	–	I
*Ocotea pluridomatiata*	GQ480389.1	–	–	I
*Ocotea pomaderroides*	GQ480390.1	MK507352.1	–	I
*Ocotea porosa*	KF420956.1	KF421040.1	OM135251.1	I
*Ocotea porphyria*	MF110079.1	MF138004.1	–	I
*Ocotea praetermissa*	KX509872.1	KX509934.1	–	I
*Ocotea puberula*	KF420951.1	KF421045.1	–	I
*Ocotea pulchella*	KX509873.1	KX509935.1	–	I
*Ocotea purpurea*	KX509874.1	KX509936.1	–	I
*Ocotea racemosa*	MK507283.1	MK507354.1	–	I
*Ocotea ramosissima*	GQ480393.1	–	–	I
*Ocotea rivularis*	MK507284.1	MK507355.1	–	I
*Ocotea salvadorensis*	KX509875.1	KX509938.1	–	I
*Ocotea sambiranensis*	MN431707.1	MN431734.1	–	I
*Ocotea sassafras*	MK507285.1	MK507356.1	–	I
*Ocotea schomburgkiana*	AF272315.1	–	–	I
*Ocotea sessiliflora*	MN431708.1	MN431735.1	–	I
*Ocotea silvestris*	GQ480394.1	–	–	I
*Ocotea sinuata*	KX509876.1	KX509939.1	–	I
*Ocotea spectabilis*	MK507287.1	MK507358.1	–	I
*Ocotea spixiana*	AF272316.1	–	–	I
*Ocotea tabacifolia*	–	–	OM135252.1	I
*Ocotea teleiandra*	MK507288.1	MK507359.1	–	I
*Ocotea tenera*	MF110082.1	MF138006.1	–	I
*Ocotea tessmannii*	MK507290.1	MK507361.1	–	I
*Ocotea thouvenotii*	MN431709.1	MN431736.1	–	I
*Ocotea tomentella*	AF272317.1	AF268765.1	–	I
*Ocotea trichantha*	MN431710.1	MN431737.1	–	I
*Ocotea trichophlebia*	MN431711.1	MN431738.1	–	I
*Ocotea tristis*	AF272318.1	–	–	I
*Ocotea valeriana*	MK507292.1	MK507363.1	–	I
*Ocotea velloziana*	GQ480395.1	–	–	I
*Ocotea venulosa*	MN431712.1	MN431739.1	–	I
*Ocotea whitei*	MK507286.1	MK507357.1	–	I
*Ocotea zahamenensis*	MN431713.1	MN431740.1	–	I
*Paraia bracteata*	MK507293.1	MK507364.1	–	I
*Persea americana*	AF272322.1	–	NC_031189.1	Outgroup
*Phoebe sheareri*	FM957849.1	–	NC_031191.1	Outgroup
*Pleurothyrium cinereum*	AF272329.1	AF268769.1	–	I
*Pleurothyrium cuneifolium*	KX509879.1	KX509941.1	–	I
*Pleurothyrium insigne*	AF272330.1	–	–	I
*Pleurothyrium poeppigii*	KX509880.1	KX509942.1	–	I
*Pleurothyrium trianae*	MK507294.1	MK507365.1	–	I
*Rhodostemonodaphne capixabensis*	GQ480398.1	–	–	I
*Rhodostemonodaphne crenaticupula*	AF272331.1	AF268759.1	**–**	I
*Rhodostemonodaphne kunthiana*	AF363384.1	FJ038959.2	**–**	I
*Rhodostemonodaphne negrensis*	MK507295.1	MK507366.1	**–**	I
*Rhodostemonodaphne parvifolia*	AF363386.1	MK507367.1	**–**	I
*Rhodostemonodaphne peneia*	AF363387.1	–	**–**	I
*Rhodostemonodaphne praeclara*	AF272332.1	AF268760.1	–	I
*Rhodostemonodaphne recurva*	AF272333.1	–	–	I
*Rhodostemonodaphne scandens*	AF272334.1	–	–	I
*Sassafras albidum*	EF491214.1	AF268793.1	–	III
*Sassafras randaiense*	EF491212.1	EF491222.1	MW337246.1	III
*Sassafras tzumu*	AF272336.1	EF491220.1	NC_045268.1	III
*Umbellularia californica*	AF272337.1	AF268777.1	–	I
*Urbanodendron bahiense*	MK507296.1	MK507368.1	–	I
*Urbanodendron verrucosum*	MK507297.1	MK507369.1	–	I
